# Differential Effects of Angiotensin-II Compared to Phenylephrine on Arterial Stiffness and Hemodynamics: A Placebo-Controlled Study in Healthy Humans

**DOI:** 10.3390/cells10051108

**Published:** 2021-05-05

**Authors:** Klaas F. Franzen, Moritz Meusel, Julia Engel, Tamara Röcker, Daniel Drömann, Friedhelm Sayk

**Affiliations:** 1 Medizinische Klinik III, Universitätsklinikum Schleswig-Holstein, 23538 Lübeck, Germany; daniel.droemann@uksh.de; 2 Airway Research Center North, Member of the German Center for Lung Research (DZL), 35392 Gießen, Germany; 3 Medizinische Klinik II, Universitätsklinikum Schleswig-Holstein, 23538 Lübeck, Germany; moritz.meusel@uksh.de; 4 Medizinische Klinik I, Universitätsklinikum Schleswig-Holstein, 23538 Lübeck, Germany; Jul.Engel@web.de (J.E.); roecker-tamara@web.de (T.R.); friedhelm.sayk@uksh.de (F.S.)

**Keywords:** α_1_-adrenoceptor, phenylephrine, Angiotensin II, baroreflex, arterial stiffness, augmentation index, pulse wave velocity

## Abstract

The α_1_-adrenoceptor agonist phenylephrine (PE) and Angiotensin II (Ang II) are both potent vasoconstrictors at peripheral resistance arteries. PE has pure vasoconstrictive properties. Ang II, additionally, modulates central nervous blood pressure (BP) control via sympathetic baroreflex resetting. However, it is unknown whether Ang II vs. PE mediated vasoconstriction at equipressor dose uniformly or specifically modifies arterial stiffness. We conducted a three-arm randomized placebo-controlled cross-over trial in 30 healthy volunteers (15 female) investigating the effects of Ang II compared to PE at equal systolic pressor dose on pulse wave velocity (PWV), pulse wave reflection (augmentation index normalized to heart rate 75/min, AIx) and non-invasive hemodynamics by Mobil-O-Graph™ and circulating core markers of endothelial (dys-)function. PE but not Ang II-mediated hypertension induced a strong reflex-decrease in cardiac output. Increases in PWV, AIx, total peripheral resistance and pulse pressure, in contrast, were stronger during PE compared to Ang II at equal mean aortic BP. This was accompanied by minute changes in circulating markers of endothelial function. Moreover, we observed differential hemodynamic changes after stopping either vasoactive infusion. Ang II- and PE-mediated BP increase specifically modifies arterial stiffness and hemodynamics with aftereffects lasting beyond mere vasoconstriction. This appears attributable in part to different interactions with central nervous BP control including modified baroreflex function.

## 1. Introduction

Arterial hypertension is one of the strongest risk factors for cardiovascular morbidity and mortality. Its pathophysiology involves the stiffening of central elastic large arteries, an increase in total vascular resistance at peripheral arteries and endothelial dysfunction. Arterial stiffness is a generic term describing the structural and functional properties of the arterial vascular tree. In brief, the pulse pressure wave, primarily caused by cardiac contraction, increases from the aorta to peripheral arteries. This originates from progressively reduced elasticity of the vessel wall combined with reduced vessel diameters from the aorta to peripheral arteries. The pulse wave is reflected from peripheral resistance arteries, which augments the central aortic blood pressure (BP). Therefore, arterial stiffness depends on both central elastic and peripheral vascular wall tension and can be characterized via measurements of antegrade pulse wave velocity (PWV) and the augmentation index of the reflected pressure wave [1,2]. This index depends on heart rate (HR) [3], and therefore, by convention, the augmentation index is adjusted to a HR of 75 bpm to obtain a normalized and HR independent measure—AIx.

Hypertension increases aortic stiffness and PWV, which further enhances the augmentation of the reflective retrograde pulse wave and increases central aortic BP. Thus, aortic pulse pressure (PPao) is an indirect indicator of central aortic stiffness. Atherosclerotic changes contribute to chronic arterial stiffening, and, therefore, the measurement of arterial stiffness is a surrogate marker of vascular aging as well as predictive for future cardiovascular events and is of greater importance than any single measurement of BP [4]. Consistently, current European Society of Cardiology (ESC)/European Society of Hypertension (ESH) and American Heart Association (AHA) hypertension guidelines recommend measuring aortic BP as well as PWV and AIx at rest for the assessment of subclinical end-organ damage [2,5]. On the other hand, arterial stiffness is subject to considerable acute variability mediated by short-acting vasodilator/-constrictor signals. Peripheral vasoconstriction enhances pulse wave reflection with short-term changes in pulse-pressure augmentation. Concomitantly, acute BP changes alter the wall-tension of central elastic arteries, and, therefore, both PWV and AIx depend on the prevailing BP and vasoconstrictive tone rather than cardiac systolic function. Additionally, acute stressful stimuli on the vessel wall modify endothelial function, which again influences arterial stiffness [6,7].

In order to discriminate the pure impact of vasoconstrictive BP increase on arterial stiffness, we choose to compare two well-known vasoactive principles at equal hypertensive systolic target BP in healthy humans: (1) Angiotensin II (Ang II), a G-protein stimulator at vascular AT_1_-receptors [8], and (2) α_1_-adrenergic stimulation with phenylephrine (PE). They both exert potent short-lasting vasoconstriction, but it is unknown whether Ang II mediated vs. α_1_-adrenergic vasoconstriction uniformly or specifically modifies arterial stiffness. PE has purely peripheral action and, therefore, the PE-mediated BP increase is answered by reflex-bradycardia and decreased cardiac output. In contrast, Ang II permeates across the blood–brain barrier into hypothalamic and brain stem structures and adjusts the BP setpoint of the sympathetic baroreflex loop to accept higher BP—a process termed resetting [9]. Thus, BP elevation mediated by Ang II is accompanied by a largely attenuated baroreflex-response as compared to the strong counter-regulatory sympathoinhibition induced by equipotent peripherally acting vasoconstrictors such as PE [10,11,12,13]. This might alter hemodynamics at large elastic (“aortic”) as well as peripheral resistance arteries differentially. Moreover, it is unclear whether the acute changes in arterial stiffness and aortic hemodynamics in response to altered vasoconstrictor commands would rapidly return to baseline level or might have prolonged aftereffects, when the vasoconstrictors are washed-out and unaffected resting conditions are restored.

In the present human study, we investigated non-invasive markers of arterial stiffness (PWV, AIx) as well as functional parameters indicative of central (large elastic arteries), termed “aortic”, and peripheral (resistance arteries) hemodynamics before, during, and after hypertensive BP elevation mediated by the infusion of Ang II, compared to α_1_-adrenergic vasoconstriction via the infusion of PE at equipressor dose, and to a normotensive control condition (placebo). We hypothesized that PWV and AIx would significantly increase compared to placebo but not differ substantially between both vasoconstrictors at similar systolic BP elevation. Aortic and peripheral hemodynamics, however, would mirror substance-related differential effects on baroreflex function. Moreover, we compared potential prolonged or rebound changes after stopping infusion and washout of the respective vasoconstrictors.

## 2. Materials and Methods

### 2.1. Study Cohort and Design

This single-center study included 32 young, healthy normotensive, male and female (n = 16) volunteers (mean ± SD; 24.5 ± 2.6 years, 71.8 ± 9.2 kg, 22.6 ± 1.9 kg·m^−2^). All participants were screened for the following exclusion criteria: (i) mental disorders, (ii) smoking, (iii) thyroid disease, (iv) diabetes mellitus, (v) abnormal physical examination, (vi) arterial hypertension, or (vii) pregnancy. None of the volunteers had worked on night shifts for the last 2 weeks and abstained from alcohol or caffeinated beverages for at least 24 h prior to the experiments. Experimental sessions were separated by at least 48 h and were performed at the same time of day to avoid confounding circadian rhythm effects. In female subjects, experiments were performed in the first half of their ovulatory cycle. Participants were asked to follow the guidelines for measuring arterial stiffness [10,14] and gave written informed consent. The study was approved by the ethics committee of the University of Luebeck, Germany (protocol code 15–190, date of approval 10/09/2015).

The three different study arms consisted of (a) phenylephrine (PE, 1%; Baxter Healthcare Corporation, Deerfield, IL, USA), (b) angiotensin II (Ang II) (Angiotensin II-Acetat, Clinalfa/Bachem, Bubendorf, Switzerland) and (c) control condition (normal saline). Participants were blinded for the respective condition, and the sequence of conditions was balanced. Each experimental session comprised the following recording periods: (i) “baseline” recordings prior to infusion; (ii) “infusion period” of Ang II or PE with two step-up doses titrated to a systolic BP level of 140–150 mmHg (“step 1”) and 150–160 mmHg (“step 2”) vs. control, respectively; and (iii) the “post-infusion period” after stopping the infusion to titrate the respective vasoconstrictor infusion rate.

### 2.2. Study Protocol

The participants were investigated at our neurophysiology laboratory in a relaxed semi-supine position with normal room temperature, illumination and humidity. The room was shielded from external stressful insults. Oscillometric BP was monitored conventionally at the upper arm throughout the session with an automated cuff (Vital Signs Monitor Serie 300™, Welch Allyn, Skaneateles Falls, NY, USA) to titrate the respective vasoconstrictor infusion rate in order to establish and maintain a steady state at target systolic BP during the infusion period. The infusion protocol was stopped in case of prolonged reflex bradycardia below 35 heartbeats/min or if any kind of hypertensive clinical symptom occurred.

Measurements of peripheral and aortic BP and parameters of arterial stiffness were performed on the other upper arm every 5 min throughout experimental sessions with the validated Mobil-O-Graph™ device (I.E.M. GmbH, Stollberg, Germany). Like many other devices, the Mobil-O-Graph uses the oscillometric technique with a standard BP cuff at the brachial artery and software algorithms (version HMS CS 4.2) as reported elsewhere [15,16,17]. Next to PWV and AIx, the device reports aortic and peripheral BP, pulse pressure (PP) as well as HR. Additionally, it also calculates cardiac stroke volume (SV) and total vascular resistance (TVR).

The protocol started with a 30-min run-in phase to accommodate. Three recordings served for “baseline” measurement prior to infusion. This was followed by the “infusion period” which contained two incremental dose steps. Each 60-min dose step started with a 30-min titration phase in order to adjust infusion rates to a brachial systolic target BP of 140–150 mmHg (step 1) or 150–160 mmHg (step 2). This was followed each by a 30-min phase for analysis while the target BP was maintained at steady-state by further dose-adjustment as needed (“step 1” and “step 2”). Then, after stopping the respective infusion and awaiting a 30-min washout phase, recordings were taken for another 30 min as the “post-infusion period”.

### 2.3. Biochemistry

Blood samples were taken on ice from an indwelling antecubital venous catheter after 30 min of supine rest prior to infusion and at the post-infusion period about 30 min after stopping the infusion. Additionally, blood samples were drawn for measurements of Ang II and markers of endothelial function at the end of infusion “step 1” and “step 2”. Ang II sample tubes were prepared with Bestatin to stabilize. Blood samples were immediately centrifuged for 10 min at 4 °C, aliquoted and the plasma or serum stored at −80 °C until assay. Plasma/serum renin, Ang II, cortisol, aldosterone, Endothelin-1, high sensitivity C-reactive protein (hs-CRP), monocyte chemoattractant protein 1 (MCP-1) and soluble vascular cell adhesion molecule 1 (sVCAM-1) were determined using commercial assays. Serum electrolytes were measured according to routine laboratory methods.

### 2.4. Data Analysis

**Figure 1:**
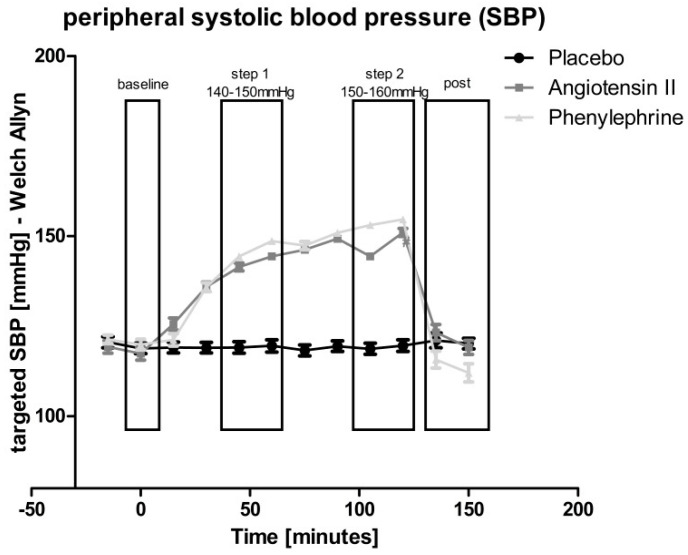
Oscillometric brachial systolic blood pressure (SBP) readings for dose titration of Angiotensin II (Ang II) (dark-grey) or phenylephrine (PE) (light-grey) vs. placebo (black). SBP was almost equivalent between both vasoconstrictors as intended, and was maintained at target level of about 140(–150) mmHg (step 1) and 150(–160) mmHg (step 2) during the respective 30-min steady-state recording periods.

Mean values of repeated measurements during the prior-to-infusion period were taken for statistical reference (“baseline”) of hemodynamics as well as arterial stiffness. Throughout the whole observation period, Mobil-O-graph measurements were performed at 5-min intervals, and three subsequent 5-min measurements were averaged to obtain means for 15-min intervals at each recording period. Next to “baseline” measurements prior to infusion, we analyzed 30-min periods at steady-state after dose titration of “step 1” (systolic BP 140–150 mmHg) and “step 2” (systolic BP 150–160 mmHg), and after a 30-min washout phase from 30–60 min after stopping the infusion (“post-infusion”) for each experimental condition, respectively, as depicted in Figure 1.

All data were checked for normal distribution by Kolmogorov–Smirnov tests. ANOVA was calculated for within-subject comparison between different recording periods of each experimental condition (repeated measures factor “time”) as well as for the comparison of corresponding recording periods between the different experimental conditions (repeated measures factor “treat”) and finally the “treat x time” interaction. Post hoc analysis was performed via Bonferroni test. A paired Student’s *t*-test and Wilcoxon test with the “baseline” period as reference were used to analyze biochemical parameters as appropriate. If not otherwise stated, all data are expressed as mean±standard error of mean (SEM). A Greenhouse–Geisser corrected *p*-value of <0.05 was considered statistically significant. Statistical analyses were performed with SPSS statistical software (SPSS 23 Inc., Chicago, IL, USA). Graphs were edited and prepared for publication with GraphPad Prism 5.0 (GraphPad Software Inc., San Diego, CA, USA). Additional labelling of the graphs was prepared using Corel Draw 11.0 (Corel Inc., Mountain View, CA, USA).

## 3. Results

### 3.1. Baseline Characteristics and Participant Drop-Outs

Two experimental phenylephrine sessions had to be stopped due to persistent bradycardia < 35 heartbeats/min. According to our predefined exclusion criteria, the participants were excluded from further statistical analysis, resulting in a total sample size of 30 volunteers (15 female) who completed all conditions.

### 3.2. Hemodynamics

**Figure 2:**
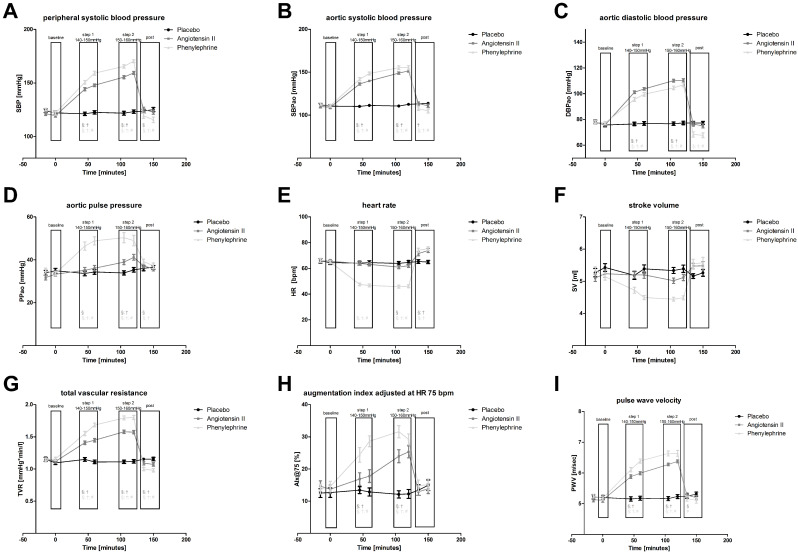
Hemodynamic parameters and arterial stiffness throughout the experiments consisting of baseline, infusion step 1, step 2 and post-infusion period. (**a**) peripheral systolic blood pressure (SBP), (**b**) aortic systolic blood pressure (SBPao), (**c**) aortic diastolic blood pressure (DBPao), (**d**) aortic pulse pressure (PPao), (**e**) heart rate (HR), (**f**) stroke volume (SV), (**g**) total vascular resistance (TVR), (**h**) augmentation index at HR 75 bpm (AIx), (**i**) pulse wave-velocity (PWV). Values are expressed as means±standard error of mean (SEM). Ang II (dark grey) or PE (light grey) vs. placebo (black). Comparison of different periods within condition (ANOVA) § *p* < 0.05. Comparison of corresponding periods between conditions (ANOVA) † *p* < 0.05 for Ang II or PE vs. placebo, # *p* < 0.05 for Ang II vs. PE.

Overall ANOVA found that all three experimental conditions differed significantly during the course of each session (factor “treat x time” interaction) with regard to all of the hemodynamic characteristics that were analyzed. However, according to the predefined recording periods (i.e., “baseline” prior to infusion, infusion “step 1” and “step 2”, and “post-infusion” period), this analysis needs a more detailed description. Hemodynamic parameters are reported in detail in Table 1 and Table 2 and depicted in Figure 2a–i.

At “baseline” prior to infusion, aortic and peripheral BP, PPao, TVR, HR and SV did not show any differences between experimental conditions. This indicates that hemodynamic baseline characteristics were highly reproducible between all three sessions (Table 1 and Table 2).

At the “infusion” period, Ang II or PE infusion rates were successfully titrated to equivalent systolic BP as intended, and this was maintained at a target level of 140–150 mmHg (step 1) and 150–160 mmHg (step 2) during the respective 30-min steady-state recording periods (Figure 1).

This was accompanied by a clear increase in total vascular resistance (TVR), which was stronger during PE infusion as compared to Ang II (*p* < 0.05). Systolic BP elevation via PE but not via Ang II infusion induced significant reflex-bradycardia combined with decreased stroke volume (SV), leading to lower diastolic aortic and peripheral BP during PE infusion as compared to Ang II. This resulted in a significantly stronger increase in pulse pressure (PPao and PPp) during the PE infusion steps 1 and 2. Still, PPao also progressively increased with Ang II, attaining statistical significance at infusion “step 2” (+11% from baseline, *p* < 0.05).

At the “post-infusion” period, aortic and peripheral systolic and diastolic BP rapidly declined following both vasoconstrictors, and, following Ang II, did not differ from the “post-infusion” placebo level. Following PE, in contrast, aortic and peripheral systolic and diastolic BP decreased even below the “baseline” level (diastolic −9%, *p* < 0.05), and was significantly lower as compared to the corresponding Ang II or placebo “post-infusion” data (*p* < 0.05). TVR decreased below baseline or placebo level following both vasoconstrictors, but this was significantly stronger after PE. PPao and PPp declined but were still higher than the respective baseline (both vasocontrictors) or corresponding placebo level (PE). HR and SV significantly increased beyond respective “baseline” or corresponding placebo levels (*p* < 0.05) following both vasoactive drugs (Ang II *p* < 0.01; PE *p* < 0.01).

### 3.3. Parameters of Arterial Stiffness

Overall ANOVA on PWV and AIx was highly significant for the repeated measures factors “treatment”, “time” and “treat x time interaction”. At baseline prior to infusion PWV and AIx normalized to HR 75/min were almost identical in all three study arms. During the infusion of PE or Ang II, PWV and AIx significantly increased as compared to baseline and to the corresponding placebo infusion period. This increase in both PWV and AIx appeared to be dose-dependent and was stronger at the PE condition (max. about +100% from baseline, *p* < 0.001) compared to Ang II (max. about +74% from baseline, *p* < 0.001). At the “post-infusion” period, PWV and AIx rapidly returned to respective baseline levels following either vasoactive drug and did no longer differ from corresponding placebo data.

### 3.4. Biochemistry

Data of laboratory parameters are presented in detail in Table 3 and in the supplemental table. In brief:

#### 3.4.1. Electrolytes

Serum electrolytes did neither differ between experimental conditions at the baseline prior to infusion nor during the infusion “step 1”, “step 2” or post-infusion period. Likewise, there were no significant changes during the course of the respective experimental sessions.

#### 3.4.2. Hormones

Serum cortisol levels significantly decreased by 59% (*p* < 0.01) at the placebo condition from “baseline” until the “post-infusion” period, while cortisol levels did not change significantly during the Ang II and PE condition. Within-subject comparison between the three conditions revealed a significant difference at “post-infusion” (*p* < 0.01).

During Ang II infusion, serum aldosterone levels significantly increased by 21% from “baseline” to “post-infusion” (*p* < 0.05). In contrast, the aldosterone level decreased significantly between these two time points during PE (−27%; *p* < 0.01) and placebo infusion (−19%, *p* < 0.01). The ANOVA and post hoc analysis across the three conditions revealed significant differences at “post-infusion” (*p* < 0.01).

Renin significantly decreased by 31% (*p* < 0.05) during Ang II, but did not change during PE or placebo infusion. Levels were significantly lower following Ang II compard to PE.

As intended, angiotensin II plasma levels sharply increased up to 5-fold during Ang II infusion and rapidly returned near “baseline” at the “post-infusion” period. Angiotensin II plasma levels did not change, in contrast, at the PE or placebo condition at any time. The Ang II infusion led to significant differences between experimental conditions after “step 1” and after “step 2” but not at “post-infusion”.

#### 3.4.3. Inflammation and Endothelial Dysfunction

Endothelin-1 significantly increased at “step 1” in all three infusion regimes. At “step 2”, Endothelin-1 levels were significantly higher during Ang II or PE infusion compared to placebo condition. HsCRP and sVCAM-1 did not show any differences within or between conditions. MCP-1 significantly increased after “step 1” and “step 2” of Ang II or PE infusion. This increase was significantly stronger during PE infusion compared to Ang II. Overall ANOVA and respective post hoc analysis revealed significant differences between the three conditions at infusion “step 1” and “step 2”.

### 3.5. Analysis of Gender Effects

Subgroup analysis of male vs. female participants did not reveal any gender-related differences in hemodynamics, arterial stiffness, biochemistry or parameters of endothelial function at any period of any of the three experimental conditions.

## 4. Discussion

The purpose of our study was to discriminate the pure impact of (vasoconstrictory) BP increase on arterial stiffness in a systemic physiological approach. We investigated whether two different vasoconstrictor pathways with different baroreflex responses uniformly or specifically modify PWV and AIx. Evidently, vasoconstriction mediated by AT_1_-receptors vs. α_1_-adrenoceptors had different effects on arterial stiffness as well as on aortic and peripheral hemodynamics at equal systolic pressor doses recorded non-invasively with the validated Mobil-O-Graph™. Moreover, we found prolonged aftereffects at the “post-infusion” period, suggestive of different rebound phenomena and/or prolonged baroreflex resetting which lasted beyond the withdrawal and washout of either vasoactive infusion.

### 4.1. Arterial Stiffness and Hemodynamics during Vasoactive Drug Infusion

Ang II mediated hypertension did not induce any obvious baroreflex counter-regulation. Physiologically, changes in BP of this magnitude as in our experiments (step 1 and step 2) should be vigorously counterbalanced by baroreflex-mediated increase or decrease in cardiac function (HR and SV) and in vasoconstrictive sympathetic outflow to the muscle vascular bed of resistance arteries—unless the reference BP level (setpoint) of this reflex-loop is altered. The threshold of the baroreflex feedback-loop can be readjusted to a new BP level via superordinate feed-forward signals to instantaneously address altered needs—a process called “resetting”. Our findings indicate that circulating Ang II resets the central nervous baroreflex threshold to accept higher BP with prolonged aftereffects beyond the immediate effects of the circulating hormone [9,18].

As expected, PE infusion induced reflex-bradycardia. This was accompanied by decreased cardiac SV and a significantly stronger increase in TVR compared to Ang II. The prevailing aortic mean BP was identical between both conditions. According to a simplified model, aortic BP depends on cardiac stroke, TVR and pressure augmentation of the reflected pulse wave. The decrease in cardiac output (CO=HRxSV) during PE was successfully counterbalanced by the titration of the vasoconstrictive infusion at the predefined systolic target BP (dose step 1 and 2), leading to higher TVR and higher AIx. Concomitantly, diastolic BP was lower compared to Ang II despite higher TVR, which is attributable to the prolonged diastole at reflex-bradycardia. As a result of decreased diastolic BP combined with increased AIx, the PPao was larger. In contrast to such negative chrono- and inotropic response via the baroreflex-feedback loop during PE infusion [19], CO did not change during Ang II infusion in our study. Vingerhoedt et al. previously reported that CO even increased and made a major contribution to the increase in BP response to intravenous Ang II in normotensive volunteers [20]. Moreover, they found that both vasoconstriction and increased CO were effectively blocked by the selective AT_1_-receptor antagonist telmisartan.

Ang II infusion and PE both increased PWV and AIx. In general, PWV and AIx correspond well to the prevailing BP. However, the increases in PWV and AIx were stronger during PE infusion compared to Ang II despite almost identical mean aortic BP. Elevated AIx mirror both faster aortic pulse wave propagation as well as enhanced peripheral pulse wave reflection. The higher AIx normalized to a HR of 75/min [3] during PE infusion compared to Ang II is in accordance with the concomitantly stronger elevation of TVR. Vice versa, during Ang II infusion, the attenuated increase in AIx despite equal aortic mean BP corresponds to the concomitantly blunted baroreflex decrease in CO as compared to PE. This challenges the concept that AIx appear less dependent from cardiac systolic function and suggests that arterial stiffness in healthy humans has to be interpreted in a wider integrative regulatory context.

In a clinical context, antihypertensive treatment with Ang II inhibitors substantially reduces arterial stiffness and BP [21]. Clinical studies have shown that selective α1-adrenoceptor blockade with doxazosin did not reduce PWV to the same extent as Angiotensin-converting-enzyme (ACE) inhibition with ramipril [22], while both antihypertensive drugs similarly reduced central BP after 12 weeks of treatment. Moreover, the reduction in arterial stiffness (PWV) in hypertensive subjects following ramipril was unrelated to the extent of BP reduction [4]. Therapeutic ß-receptor blockade was associated with decreased CO, but did not reduce aortic BP and even had detrimental effects on arterial stiffness, although the peripheral BP was effectively decreased. The underlying mechanisms for this difference beyond BP lowering in hypertensive patients have not yet been fully clarified. Possible explanations highlight the protective long-term effects of ACE inhibitors beyond BP reduction on structural vascular remodeling. This includes the lack of hypertrophy and fibrosis, and positive effects on collagen and elastin architecture as well as on endothelial function [12,13,22]. This long-term context, however, is not suitable for our present acute vasoconstrictor model.

### 4.2. Arterial Stiffness and Hemodynamics after Stopping Vasoactive Drug Infusion

Physiologically, the short-term changes in arterial stiffness in response to altered sympathetic or humoral vasoconstrictor commands rapidly return to baseline, as soon as unaffected resting conditions are restored. Accordingly, PWV and AIx at the “post-infusion” period had returned to pre-infusion levels. These present findings are in accordance with our previous preliminary results in normotensive human volunteers [9]. Animal studies and our previous work, however, suggested that prolonged low-dose infusions of Ang II or PE had long-lasting consequences on central nervous BP control. In the present study, the infusion of Ang II or PE had substance-specific prolonged effects at the “post-infusion” period about 30–60 min after stopping the respective vasoactive infusion compared to placebo. Following Ang II, BP rapidly returned to baseline level. Following PE, BP and TVR were decreased even below pre-infusion level and were significantly lower compared to Ang II or placebo. Concomitantly, CO (i.e., SV and HR) was increased after the withdrawal of either vasoconstrictor as compared to placebo or prior to infusion “baseline”. This suggests that the withdrawal of PE induced hypotension, which led to a chronotropic and inotropic increase in cardiac function most likely via the mere baroreflex-feedback loop. The underlying mechanism cannot be clarified with the present study design, but the concept of rebound loss of vascular tone due to down-regulation of α-adrenoceptors warrants further investigation. The significant increase in SV and HR at the withdrawal of prolonged Ang II infusion, in contrast, was combined with slightly higher systolic BP compared to prior to infusion “baseline”. This might unravel prolonged aftereffects of baroreflex resetting towards higher BP during the antecedent Ang II infusion period. In a previous study that used microneurographic techniques to directly record vasoconstrictive sympathoneural signals, the continuous infusion of Ang II in wake resting subjects (6 h, at borderline-hypertensive BP) was able to sustainably reset sympathetic baroreflex function. Such resetting led to higher BP about 1–2 h after stopping the Ang II infusion compared to placebo, while baroreflex sensitivity (i.e., the slope of sympathetic reflex-response to a vasodilatory of –constrictive stimulus) was preserved. This effect lasted far beyond the direct peripheral vasoconstrictive effect of circulating Ang II [9]. Indeed, the vasoactive forms of both PE and Ang II have a very short plasma half-life, and their vasoconstrictive action at vascular smooth muscle cells fades within few minutes. In our present study, the “post-infusion” period started after a 30-min washout period, and direct peripheral Ang II effects cannot explain our present findings.

### 4.3. Ang II—Sympathetic Baroreflex Interactions

There is an intimate crosstalk between the sympathetic baroreflex system and Ang II. The anatomical structures, cellular components and molecular mechanism involved have been reviewed in detail elsewhere [9,18]. In brief, the network of sympathetic BP control, setting the background level of vasoconstrictive sympathetic outflow, primarily involves the hypothalamus and the brain stem. Core structures such as the paraventricular nucleus (PVN), the rostral ventrolateral medulla (RVLM), and the nucleus of the solitary tract (NTS) are modulated by Ang II. In animal models, intracerebral Ang II administration into the PVN or RVLM increased BP and sympathetic nerve activity. These effects are abolished by ACE inhibitors or AT_1_ receptor blockers. The stimulation of brain AT_2_-receptors, in contrast, might induce opposite effects on sympathetic activation [18]. Circulating Ang II can bypass the blood–brain barrier via circumventricular organs which project to these nuclei [23]. Additionally, circulating Ang II reduces, e.g., the transmission between baroreceptor afferents and NTS efferent neurons by activating endothelial AT_1_ receptors, which causes the release of nitric oxide. Nitric oxide, which is freely diffusible, subsequently crosses the blood–brain barrier and potentiates gamma-Aminobutyric acid (GABA) release. GABA-mediated inputs can bias the response of NTS second-order neurons to baroreceptor afferent stimulation, leading to a resetting of the baroreflex toward a higher BP level. In humans, bare vasoconstrictive sympathetic outflow is increased when the pressor effects of Ang II infusion were neutralized by simultaneous infusions of the vasodilator nitroprusside [24,25]. The effect of ANG II on RVLM-barosensitive neurons seems to rely on several mechanisms that could interact, including the closure of a resting potassium conductance located on the barosensitive neurons, reactive oxygen species and modified gene expression. Hypothalamic projections are the most relevant feed-forward signals for resetting baroreflex pathways at the brain stem. However, baroreceptors also exert powerful influences on the hypothalamus and beyond and, therefore, abnormalities of baroreceptor afferent input, or of its processing in the NTS, could rely on mechanisms that are much more complex than a simple brain stem reflex dysfunction. Projections between baroreceptor afferents and the hypothalamus could influence sodium and volume regulation, including ANG II-mediated control of these mechanisms such as vasopressin release. Moreover, increased production of intracellular radical oxygen species specifically in the subfornical organ seems to be crucial to the development of neurogenic hypertension mediated by inappropriately high levels of circulating Ang II. Consistently, therapeutic blockade of elevated Ang II levels in hypertensive patients substantially decreases BP without any baroreflex-mediated sympathoactivation and might even reverse sympathetic hyperactivity, indicating a central nervous downward resetting of the sympathetic baroreflex setpoint.

### 4.4. Biochemistry and Markers of Endothelial Function

In the present study, the plasma level of circulating renin and serum aldosterone changed during Ang II infusion as physiologically expected. During placebo or PE infusion, in contrast, Ang II was unchanged and aldosterone slightly decreased. In the placebo condition, cortisol levels declined as expected from its well-known circadian rhythm, but remained unchanged during the PE and Ang II condition. However, the differences were small and the schedule of our experiments was too short to expect hemodynamically relevant changes in fluid balance. Likewise, vascular remodeling due to elevated aldosterone following Ang II infusion is unlikely to occur within this timeframe.

Impaired endothelial function and vascular microinflammation play an important role in cardiovascular risk. Acute stressful stimuli on the vessel wall modify endothelial function, which again influences arterial stiffness. As a nested pilot approach, we assessed four markers of endothelial dysfunction which are indicative of microinflammation, vascular cell adhesion and vasoconstriction. Endothelin-1 (ET-1) was significantly increased with both Ang II and PE. ET-1 is released from endothelial cells in response to various stimuli including shear stress. Mainly, it is a potent vasoconstrictor and mitogen via G-protein coupled ET_A_ receptors at smooth muscle cells and contributes to early atherosclerosis. Experimental data suggested that Ang II may contribute to elevated levels of circulating ET-1. We observed an increase in MCP-1 during the infusion of Ang II and PE. This is in accordance with known chronic effects in human arterial hypertension, since an elevated level of MCP-1 is associated with an increased risk of atherosclerosis, ranging from subclinical changes to overt myocardial infarction [26]. Pathogenetically, MCP-1 regulates the migration and infiltration of monocytes and macrophages and, therefore, can be used as a direct marker of microinflammatory activity [27]. This increase was slightly stronger during PE infusion compared to Ang II, suggesting that the increase in MCP-1 might be related to higher PPao and TVR. However, there was no substance-related difference in MCP-1 decreases after antihypertensive treatment over 12 weeks with either doxazosin or ramipril [22,26]. Apart from MCP-1, our study showed no significant effects on sVCAM and hsCRP. Taken together, our intervention induced some early changes. However, this pilot approach covered only acute effects within 2–3 h after the BP elevation—a timeframe that is short for, e.g., the signal cascade that induces hsCRP—and is not suitable to thoroughly characterize complex endothelial mechanisms in detail.

### 4.5. Limitations

Basically, systemic BP control is regulated by a complex network of integrative subsystems at various regulatory levels. Physiologic studies in healthy humans have the advantage of translating cellular and molecular research findings into a non-deterministic organism without confounding illness. Therefore, this scientific field bridges and modifies knowledge from bench to bedside. In our system approach, information about interrelations between various factors, rather than simple physiology of individual elements, is critically important. The inference of a function from its experimental modification, such as systolic BP in our study, represents a typical and modern strategy of physiologic research in humans. Therefore, any experimental model is affected by simplification. In the present study, we focused on two principle vasoconstrictive mechanisms, Ang II and α_1_-adrenergic receptor activation. Moreover, we only evaluated acute but not chronic hemodynamic responses and changes in arterial stiffness with a non-invasive device that uses validated algorithms. Our findings are rather descriptive; their interpretation relies on the selected well-established cybernetic and hemodynamic concepts described elsewhere which, however, cannot be confirmed or disproved with this human non-invasive approach [5,7]. The measurement of mechanical and neural baroreceptor sensitivity [28] and invasive measurements of hemodynamics, microneurographic recordings of vasoconstrictive muscle sympathetic nerve activity combined with pharmacologic baroreflex challenges and measurements of plasma catecholamines, as well as dynamic assessment of microcirculation and of novel markers of endothelial function, might refine the approach and warrant future investigation.

Another limitation is that the benchmark for the titration of vasoconstrictor infusion rate was brachial oscillometric systolic BP, and, thus, the dose of Ang II or PE per bodyweight was not standardized. Titration to equal TVR at the cost of different BP could alternatively suit to compare effects on arterial stiffness. An important strength of our study is the strictly controlled design permitting within-subject comparison of a defined intervention at standardized experimental circumstances. This approach reduced confounders and allowed robust conclusions of some key aspects, while others cannot be addressed with similar specificity. Finally, our gender-balanced study comprising 30 volunteers prevailed high statistical power and even permitted gender-related subgroup analysis.

## 5. Conclusions

Different vasoconstrictive pathways such as Ang II vs. PE exert differential hemodynamic changes. These modify arterial stiffness not only attributable to the BP elevation, per se. Differences are in part explained by a strong baroreflex-mediated decrease in cardiac output (HRxSV) during PE infusion in contrast to baroreflex resetting towards higher BP without any reflex-change in cardiac function during Ang II infusion. The interpretation of acute changes in arterial stiffness has to bear in mind the superordinate integrative neural control of BP.

Moreover, both vasoconstrictors had different aftereffects at the “post-infusion” period: the withdrawal of PE infusion induced rebound hypotension which led to chrono- and inotropic increases in cardiac function, most likely mediated via the baroreflex-feedback loop. At the withdrawal of Ang II infusion, in contrast, the increased cardiac output was not combined with decreased BP. This might unravel the sequelae of baroreflex resetting towards higher BP levels during the antecedent prolonged Ang II exposure.

## Figures and Tables

**Table 1:** Peripheral blood pressure (BP) with systolic (SBP), diastolic blood pressure (DBP) and pulse pressure (PP), heart rate (HR), cardiac stroke volume (SV) and total vascular resistance (TVR) of all three conditions at different recording periods. Comparison of different periods within condition (ANOVA) § *p* < 0.05. Comparison of corresponding periods between conditions (ANOVA) † *p* < 0.05 for Ang II or PE vs. placebo, # *p* < 0.05 for Ang II vs. PE.

		Placebo				Angiotensin II				Phenylephrine			
		Mean		sem	ANOVA	Mean		sem	ANOVA	Mean		sem	ANOVA
**Baseline**	SBP [mmHg]	123.1	±	1.3		120.4	±	1.0		123.4	±	1.1	
	DBP [mmHg]	75.4	±	1.0		75.7	±	0.7		76.6	±	0.8	
	PP [mmHg]	47.6	±	1.0		44.7	±	0.7		46.8	±	1.1	
	HR [bpm]	65.1	±	1.1		65.9	±	1.0		65.6	±	1.0	
	SV [mL]	5.3	±	0.1		5.2	±	0.1		5.2	±	0.1	
	TVR [mmHg × min/L]	1.12	±	0.02		1.14	±	0.02		1.15	±	0.02	
**Step 1 (140–150 mmHg)**	SBP [mmHg]	121.9	±	1.1		146.0	±	1.0	§; †	154.8	±	1.3	§; †; #
	DBP [mmHg]	75.1	±	1.0		100.8	±	0.7	§; †	94.6	±	1.1	§; †; #
	PP [mmHg]	46.8	±	0.9		45.1	±	1.1		60.2	±	1.4	§; †; #
	HR [bpm]	64.3	±	1.1		63.5	±	0.9		47.1	±	0.7	§; †; #
	SV [mL]	5.3	±	0.1		5.2	±	0.1		4.6	±	0.1	§; †; #
	TVR [mmHg × min/L]	1.13	±	0.02		1.43	±	0.02	§; †	1.62	±	0.02	§; †; #
**Step 2 (150–160 mmHg)**	SBP [mmHg]	122.5	±	1.2		157.3	±	1.0	§; †	167.8	±	1.1	§; †; #
	DBP [mmHg]	75.4	±	0.9		108.3	±	0.9	§; †	103.6	±	0.9	§; †; #
	PP [mmHg]	47.1	±	0.9		49.0	±	1.0	§	64.2	±	1.3	§; †; #
	HR [bpm]	64.3	±	1.0		61.5	±	0.9	§	45.9	±	0.8	§; †; #
	SV [mL]	5.4	±	0.1		5.1	±	0.1	†	4.5	±	0.0	§; †; #
	TVR [mmHg × min/L]	1.11	±	0.02		1.57	±	0.02	§; †	1.80	±	0.02	§; †; #
**Post**	SBP [mmHg]	124.5	±	1.3		124.1	±	1.4	§	117.5	±	1.7	§; †; #
	DBP [mmHg]	75.8	±	0.9		74.1	±	1.1	§	66.9	±	1.4	§; †; #
	PP [mmHg]	48.6	±	1.0		50.0	±	0.9	§	50.5	±	1.2	§; †
	HR [bpm]	65.1	±	1.0		72.7	±	1.1	§; †	74.7	±	1.1	§; †
	SV [mL]	5.2	±	0.1		5.5	±	0.1	§; †	5.6	±	0.1	§; †
	TVR [mmHg × min/L]	1.15	±	0.02		1.08	±	0.02	§; †	0.99	±	0.02	§; †; #

**Table 2:** Aortic blood pressure (SBPao and DBPao), pulse-wave velocity (PWV) and augmentation index adjusted to HR 75/min (AIx) of all three conditions at different recording periods. Comparison of different periods within condition (ANOVA) § *p* < 0.05. Comparison of corresponding periods between conditions (ANOVA) † *p* < 0.05 for Ang II or PE vs. placebo, # *p* < 0.05 for Ang II vs. PE.

		Placebo				Angiotensin II				Phenylephrine			
		Mean		sem	ANOVA	Mean		sem	ANOVA	Mean		sem	ANOVA
**Baseline**	SBPao [mmHg]	111.3	±	1.2		109.5	±	1.1		111.9	±	1.0	
	DBPao [mmHg]	76.8	±	1.0		76.9	±	0.8		77.5	±	0.8	
	PPao [mmHg]	34.4	±	0.8		32.5	±	0.8		34.3	±	0.8	
	AIx [%]	12.6	±	1.0		14.2	±	1.1		14.1	±	1.1	
	PWV [m/s]	5.2	±	0.0		5.1	±	0.0		5.2	±	0.0	
**Step 1 (140–150 mmHg)**	SBPao [mmHg]	110.7	±	1.1		138.0	±	0.9	§; †	145.1	±	1.4	§; †; #
	DBPao [mmHg]	76.7	±	1.0		102.4	±	0.7	§; †	97.4	±	1.1	§; †; #
	PPao [mmHg]	34.0	±	0.7		35.6	±	0.9	§	47.6	±	1.3	§; †; #
	AIx [%]	13.1	±	0.8		17.3	±	1.4	§; †	26.5	±	1.4	§; †; #
	PWV [m/s]	5.2	±	0.0		5.9	±	0.0	§; †	6.2	±	0.1	§; †; #
**Step 2 (150–160 mmHg)**	SBPao [mmHg]	111.5	±	1.0		150.2	±	1.0	§; †	155.2	±	1.6	§; †
	DBPao [mmHg]	76.9	±	1.0		110.2	±	0.8	§; †	105.7	±	1.0	§; †; #
	PPao [mmHg]	34.6	±	0.7		40.0	±	0.9	§; †	49.6	±	1.7	§; †; #
	AIx [%]	12.2	±	0.9		24.6	±	1.4	§; †	30.4	±	1.3	§; †; #
	PWV [m/s]	5.2	±	0.0		6.3	±	0.0	§; †	6.6	±	0.1	§; †; #
**Post**	SBPao [mmHg]	113.4	±	1.1		112.2	±	1.3	†	106.1	±	1.6	§; †; #
	DBPao [mmHg]	77.2	±	0.9		75.6	±	1.2		67.9	±	1.3	§; †; #
	PPao [mmHg]	36.2	±	0.7		36.5	±	0.7	§	38.2	±	0.9	§; †
	AIx [%]	14.2	±	1.2		13.4	±	0.9		14.9	±	1.3	
	PWV [m/s]	5.3	±	0.0		5.2	±	0.0	§	5.1	±	0.1	#

**Table 3:** Laboratory data at different conditions and periods. Comparison of different periods within condition (ANOVA) § *p* < 0.05. Comparison of corresponding periods between conditions (ANOVA) † *p* < 0.05 for Ang II or PE vs. placebo, # *p* < 0.05 for Ang II vs. PE.

		Placebo				Angiotensin II				Phenylephrine			
		Mean		sem	ANOVA	Mean		sem	ANOVA	Mean		sem	ANOVA
**Baseline**	Cortisol	9.2	±	0.6		8.7	±	0.8		9.1	±	0.7	
	Aldosterone	62.6	±	4.2		51.1	±	2.8		47.4	±	3.0	
	Renin	4.6	±	0.5		4.7	±	0.6		4.9	±	0.6	
	Angiotensin	7.8	±	0.9		15.0	±	2.9		8.2	±	1.4	
**Step 1 (140–150 mmHg)**	Angiotensin	10.7	±	1.4		53.8	±	7.7	§; †	9.4	±	2.1	†; #
**Step 2 (150–160 mmHg)**	Angiotensin	9.3	±	2.3		77.8	±	10.4	§; †	8.1	±	1.5	†; #
**Post**	Cortisol	4.6	±	0.4	§	7.7	±	1.0	†	8.4	±	1.0	†
	Aldosterone	47.0	±	3.1	§	68.1	±	3.7	§; †	39.3	±	2.8	§; †; #
	Renin	4.1	±	0.4		3.4	±	0.3	§	4.8	±	0.6	#
	Angiotensin	7.7	±	0.9		20.0	±	6.8		7.8	±	1.5	

## Data Availability

Please refer to suggested Data Availability Statements in section “MDPI Research Data Policies” at https://www.mdpi.com/ethics.
